# *Lactiplantibacillus plantarum* E51 protects against *Clostridioides difficile*-induced damages on Caco-2 intestinal barrier functions

**DOI:** 10.1007/s00203-022-02837-6

**Published:** 2022-05-03

**Authors:** Huey-Sheng Jeng, Tsong-Rong Yan

**Affiliations:** 1 Department of Chemical Engineering and Biotechnology, Institute of Chemical Engineering and Biotechnology, Tatung University, No. 40, Sec. 3, Zhongshan N. Rd., Taipei, 10452 Taiwan; 2 Department of Urology, Zhong-Xing Branch, Taipei City Hospital, Taipei, 10341 Taiwan

**Keywords:** Adhesion, *Clostridioides difficile*, *Lactiplantibacillus plantarum*, Monolayer integrity, Tight junction

## Abstract

*Clostridioides difficile* (*C. difficile*) infection is associated with high morbidity and mortality. This study aimed to evaluate the protective effect of *Lactiplantibacillus plantarum* E51 (*L. plantarum* E51) on *C. difficile* infection using the Caco-2 monolayer in vitro model*.* Caco-2 cells were infected with *C. difficile* in the presence/absence of *L. plantarum* E51 or *Lacticaseibacillus rhamnosus* GG (LGG). Caco-2 intestinal barrier functions, such as monolayer integrity, IL-8 secretion, and tight junction protein expression, were quantified to investigate the extent to which *L. plantarum* E51 protected against *C. difficile* infection in vitro. Furthermore, inhibition of *C. difficile* adhesion to Caco-2 cells by *L. plantarum* E51 was explored using competition, exclusion, and displacement assays. The results indicated that *L. plantarum* E51 inhibited *C. difficile* growth, ameliorated *C. difficile*-caused decrease in transepithelial/ transendothelial electrical resistance, attenuated *C. difficile*-induced IL8 secretion, and upregulated claudin-1 protein expression that was inhibited by *C. difficile*. Moreover, *L. plantarum* E51 suppressed *C. difficile* adhesion to Caco-2 cells. In conclusion, these findings demonstrated that *L. plantarum* E51 substantially protected against *C. difficile*-induced damages on intestinal barrier functions in Caco-2 cells. The probiotic potential of *L. plantarum* E51 against *C. difficile* infection warrants further investigation.

## Introduction

*Clostridioides difficile* (*C. difficile*), an anaerobic spore-forming Gram-positive rod, is a major nosocomial pathogen that can cause diarrhea, antibiotic-associated pseudomembranous colitis, sepsis, and death (Paredes-Sabja et al. 2014). Colonization of the human intestinal tract by *C. difficile* occurs after alteration of the normal gut flora by antibiotic therapy. The pathogenesis of *C. difficile* infection is driven by the actions of its two exotoxins, Toxin A and Toxin B, which disrupt tight junctions in the intestinal epithelium and then cause inflammation (Di Bella et al. 2016; Simeon et al. 2019). *C. difficile* infection also results in cytotoxicity, apoptosis and necrosis (Tinoco-Veras et al. 2017); however, its treatments are still poorly understood (Elliott et al. 2017). Since the two main antibiotic treatments for *C. difficile* infection, metronidazole and vancomycin, have a 20% risk of reinfection, non-antibiotic treatments with sustained cure rates are being investigated (Wu and Wu 2012).

The FAO/WHO defined probiotic species as live microorganisms that confer a health benefit on the host when consumed in appropriate amounts (Hill et al. 2014). A variety of probiotic microorganisms were confirmed as beneficial to human health through their support of a healthy digestive system, such as multiple species of the lactic acid bacteria *Lactobacillus* and *Bifidobacterium* (Hill et al. 2014). Notably, 261 species of the genus *Lactobacillus* (at March 2020) were reclassified into 25 genera, including *Lactiplantibacillus* and *Lacticaseibacillus* (Zheng et al. 2020). Organisms suitable for probiotic use must be generally recognized as safe for ingestion (Sanders et al. 2010), able to survive and proliferate at gastric acid pH and in medium containing bile, and able to adhere to enterocytes (Capurso 2019).

A meta-analysis concluded that the use of confirmed probiotic species resulted in a decrease in *C. difficile* infections in patients taking antibiotics (Evans and Johnson 2015). A Cochrane Review of 31 trials (8672 participants) reported that probiotics reduced the risk of *C. difficile*-associated diarrhea by 60% (Goldenberg et al. 2017). Several lines of evidence indicated the promising effects of the probiotic agent *Lacticaseibacillus rhamnosus* GG (LGG) on *C. difficile* infection (Capurso 2019). First patented for use in 1989, LGG is the most widely studied probiotic strain (Capurso 2019). However, probiotic agents for the treatment of *C. difficile* infection are still scarce (Valdés-Varela et al. 2016). Thus, the search for organisms with the potential for clinical use as probiotic species agents is ongoing.

*Lactiplantibacillus plantarum* E51 (*L. plantarum* E51) was isolated from paocai, a Chinese fermented vegetable (Chang et al. 2013). In a preliminary screening of these isolates, *L. plantarum* E51 survived at low pH and in bile and exhibited the greatest adhesion to Caco-2 cells, even greater than that of LGG (Chang et al. 2013). Caco-2 cells, an immortalized human colorectal adenocarcinoma cell line, were widely used to mimic intestinal barrier function to investigate the molecular mechanisms underlying *C. difficile* infection in vitro (Mehdizadeh Gohari et al. 2019; Ghosh et al. 2020). The aim of this study was to systematically assess the protective effect of *L. plantarum* E51 on *C. difficile-*induced damages on Caco-2 intestinal barrier functions, including monolayer integrity, IL-8 secretion, tight junction protein expression, and adhesion to Caco-2 cells. The anticipated results might provide preliminary evidence for the probiotic potential of *L. plantarum* E51 against *C. difficile* infection.

## Methods

### Bacterial strains and culture conditions

*Clostridioides difficile* of known ribotype (Ofori et al. 2018), positive for Toxins A and B (Di Bella et al. 2016) and binary toxin (Aktories et al. 2018), and LGG were obtained from the Bioresource Collection and Research Center (BCRC, Hsinchu City, Taiwan). *C. difficile* was cultured in reinforced Clostridial medium (RCM; Difco Laboratories, Franklin Lakes, NJ, USA) in an anaerobic chamber with continuous shaking at 37 °C. *L. plantarum* E51 was isolated from paocai as previously described (Chang et al. 2013). Most *Lactiplantibacillus* and *Lacticaseibacillus* species can grow under aerobic condition (Maresca et al. 2018), so both LGG and *L. plantarum* E51 were cultured in lactobacilli MRS broth (Difco Laboratories) at 37 °C in a regular orbital shaking incubator in this study. Notably, RCM was specifically designed for the cultivation and enumeration of Clostridia (Borriello and Barclay 1985). Consistently, we previously found that LGG and *L. plantarum* E51 were not able to grow in RCM, while *C. difficile* could grow in both RCM and MRS broth.

### Acid and bile salts tolerance assays

An acid tolerance assay was performed as previously described (Corcoran et al. 2005) with minor modifications. Cultures of the isolated bacteria (LGG and *L. plantarum* E51) were grown overnight in 15 mL lactobacilli MRS broth. The cultures were centrifuged at 12,000 × *g* for 5 min and washed once in an equal volume of cold phosphate-buffered saline (PBS). The pellets were re-suspended in 5 mL PBS, and the volume equivalent to approximately 10^7^–10^8^ colony-forming units (CFU)/mL was further centrifuged and re-suspended in the appropriate volume of PBS at pH 2.0 or 3.0 to mimic gastric conditions. The acidic PBS buffers were prepared by dissolving 20.214 g of Na_2_HPO_4_∙7H_2_O or 3.394 g of NaH_2_PO_4_∙H_2_O in 800 mL of water, adjusting the solution to the desired pH with HCl, and adding water to a final volume of 1000 mL. Bacteria were incubated at various pH values in a 37 °C water bath with shaking at 80 rpm for 3 h. After serial dilution and Gram staining, surviving bacteria were spread on lactobacilli MRS agar plates, and the numbers of LGG and *L. plantarum* E51 were counted and the corresponding CFU/mL was calculated.

The effect of bile salts on the growth of lactic acid bacteria cells was determined as previously described (Daniel et al. 2006) with minor modifications. Fresh cultures were inoculated into MRS broth enriched with Oxgall at three concentrations (0.2, 0.3 and 0.4%, w/v) (Sigma, St. Louis, MO, USA) and incubated at 37 °C for 12 h. Growth curves were plotted, and bile salt tolerance was determined as the percentage growth rate in bile salts, which was the ratio of (increment of OD in MRS broth with 0.2%, 0.3% or 0.4% bile salts)/(increment of OD in MRS broth without bile salts) × 100.

For gastrointestinal tract simulation, *L. plantarum* E51 was pre-treated with acid (pH 2.0 or 3.0) for 3 h, and the surviving bacteria were then collected by centrifugation (12,000 × *g*, 5 min) and washed with 0.1 M PBS (pH 7.4). The cell pellet was suspended in 3 mL MRS broth with 0.3% w/v Oxgall, and incubated at 37 °C for 12 h. After serial dilutions, the number of *L. plantarum* E51 on lactobacilli MRS agar plates was counted, and the CFU/mL was calculated. All assays were performed in triplicate.

### Growth rate of *C. difficile*

*C. difficile* (10^8^ CFU/mL) was incubated with or without in the presence or absence of LGG or *L. plantarum* E51 (10^8^ CFU/mL) in lactobacilli MRS broth for 24 h at 37 °C in anaerobic chamber. Then, 0.1 ml MRS broth containing viable *C. difficile* was spread onto RCM agar plates at 0, 6, 10, 14, and 24 h and incubated anaerobically at 37 °C. After Gram staining, the number of *C. difficile* on each RCM agar plate was counted and the corresponding CFU/mL was calculated. All experiments were performed in triplicate.

### Monolayer integrity assay

Caco-2 cells were routinely maintained in Dulbecco’s modified Eagle’s medium (DMED) supplemented with 10% fetal bovine serum (FBS), 1% non-essential amino acid, and penicillin–streptomycin (all from Sigma) in a 37 °C incubator with a humidified atmosphere of 5% CO_2_ in air. The extent of monolayer integrity was determined by measuring transepithelial/transendothelial electrical resistance (TEER). Briefly, Caco-2 monolayers were cultured on Millicell culture inserts (Merck Millipore, Burlington, MA, USA) in FBS-supplemented DMEM without antibiotics at 37 °C. Subsequently, *C. difficile* (10^8^ CFU/mL) with or without either LGG or *L. plantarum* E51 (10^8^ CFU/mL) were added to the apical compartment of Millicell cell culture inserts. TEER across the inner and outer compartments of the inserts was measured at 0, 4, 8, 12, 16, 20, and 24 h using the Millicell–ERS device (Merck Millipore).

### IL-8 ELISA assay

Caco-2 monolayers with or without *C. difficile* in the presence or absence of LGG or *L. plantarum* E51 (each 10^8^ CFU/mL), were cultured on the Millicell cell culture inserts (Merck Millipore) in FBS–supplemented DMEM without antibiotics at 37 °C. After a 15 h aerobic incubation, conditioned media from Caco-2 cells were harvested and centrifuged. The concentration of IL-8 in the supernatant was quantified using the human IL-8 ELISA kit (Thermo Fisher Scientific, Waltham, MA, USA) according to the manufacturer’s instruction.

### Western blotting

After a 15-h aerobic incubation with or without *C. difficile* in the presence or absence of LGG or *L. plantarum* E51 (each 10^8^ CFU/mL), Caco-2 cells were lysed with RIPA buffer (Thermo Fischer Scientific), and the lysate proteins were separated by SDS–PAGE and transferred to a polyvinylidene fluoride (PVDF) membrane. The membrane was blocked in a solution of 5% skim milk, 1% bovine serum albumin, and 0.1% Tween 20 in Tris-buffered saline for 1 h at room temperature. The PVDF membrane was incubated with primary antibodies against claudin-1 (1:5000 dilution; Thermo Fisher Scientific) at 4 °C overnight, followed by incubation with secondary horseradish peroxidase (HRP) conjugated goat anti-rabbit IgG (1:2500 dilution; Thermo Fisher Scientific) for 1 h at room temperature. Proteins were visualized using Pierce ECL Western Blotting Substrate (Thermo Fisher Scientific). The claudin-1 band intensity was quantified using ImageJ software (National Institutes of Health, Bethesda, MD, USA) and normalized to that of *β*-actin in the same sample.

### *C. difficile* adhesion to Caco-2 cells

Caco-2 monolayers were cultured in serum-supplemented DMEM in 24-well plates aerobically at 37 °C. Three assays for evaluating the inhibition of *C. difficile* adhesion to Caco-2 cells were conducted as previously described (Yu et al. 2011). First, competition assay: *C. difficile* (10^8^ CFU/ml) was incubated on Caco-2 monolayers in the presence or absence of *L. plantarum* E51 or LGG (10^8^ CFU/mL) for 1 h at 37 °C. Second, exclusion assay: Caco-2 monolayers were pre-incubated with *L. plantarum* E51 or LGG (10^8^ CFU/mL) for 1 h, followed by the addition of *C. difficile* (10^8^ CFU/mL) to the Caco-2 cultures and 1 h incubation at 37 °C. Third, displacement assay: Caco-2 monolayers were pre-incubated with *C. difficile* (10^8^ CFU/mL) for 1 h, followed by addition of *L. plantarum* E51 or LGG (10^8^ CFU/mL) and 1 h incubation at 37 °C. All three assays were conducted in aerobatic conditions due to the oxygen requirement of Caco-2 cells. At the end of each assay, the Caco-2 cultures were washed 3 times with sterile PBS to remove non-adherent bacteria, and were lysed with sterile distilled water for 5 min to release *C. difficile* that was adhered to Caco-2 cells. After tenfold dilution, cell lysates were spread on RCM agar plates followed by 6 h anaerobic incubation. Subsequently, the number of adhered *C. difficile* on each RCM agar plate was counted and the CFU/mL of adhered *C. difficile* was calculated. All experiments were performed in triplicate.

### Statistical analysis

CFU values are presented as the mean and standard deviation (SD). The Kruskal–Wallis test was performed to examine the differences between groups. All data are expressed as the mean ± SD. The significance level was set as two-tailed *p* < 0.05. All statistical analyses were performed using IBM SPSS statistical software version 22 for Windows (IBM Corp., Armonk, NY, USA).

## Results

### *L. plantarum* E5 survived in acidic and bile-salt media

The survival of *L. plantarum* E51 in acidic medium and 0.2% bile salts was relatively better than that of LGG (Table 1). As the most widely studied probiotic strain, the survival capability of LGG under simulated gastrointestinal conditions and in the human gastrointestinal track has been demonstrated (Dommels et al. 2009; Karu and Sumeri 2016; Capurso 2019). Since LGG served as a positive control in this study, the survival of LGG in various pH conditions was not systematically examined. The survival rate of *L. plantarum* E51 was greater at pH 3.0 than pH 2.0. The survival of *L. plantarum* E51 was not affected by the concentration of bile salt. To mimic the environment of the gastrointestinal tract, *L. plantarum* E51 was first cultured in the acidic medium (pH 3.0 or 2.0), followed by the medium containing 0.3% bile salt. Consistently, the survival of *L. plantarum* E51 was higher at pH 3.0 than pH 2.0 (Table 1).

**Table 1:** Survival of two Lactobacillus strains after exposure to acid, bile salts, and gastrointestinal tract simulation

		Lactobacillus strain	
		*L. rhamnosus* GG	*L. plantarum* E51
Acid treatment (CFU/mL)	pH 7.4 (initial)	(4.5 ± 0.71) × 10^7^	(9.8 ± 1.13) × 10^8^
	pH 3.0 (for 3 h)	(3.0 ± 0.14) × 10^5^	(6.8 ± 1.84) × 10^7^
	pH 2.0 (for 3 h)	(3.0 ± 2.83) × 10^3^	(1.4 ± 0.14) × 10^4^
Bile salt treatment	0.2% bile salt	37.86%	100%
	0.3% bile salt	NA	100%
	0.4% bile salt	NA	100%
Gastrointestinal tract simulation (CFU/mL)	pH 3.0, 0.3% bile salt	NA	(5.0 ± 1.84)×10^6^
	pH 2.0, 0.3% bile salt	NA	(1.0 ± 0.42)×10^4^

All assays were performed in triplicate

*NA* not applicable (not tested)

### *L. plantarum* E51 inhibited *C. difficile* growth

**Fig. 1:**
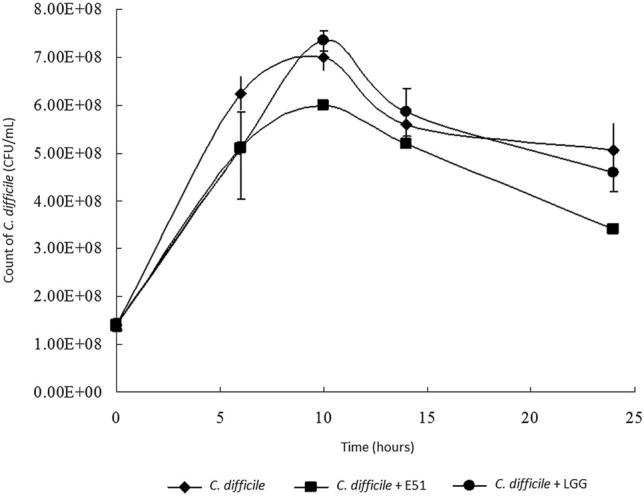
Temporal growth rates of *C. difficile* alone, or in the presence of *L. plantarum* E51 or LGG. Data are presented as the mean ± SD of triplicate samples

The growth of *C. difficile* peaked after 8–10 h of co-culture with or without *L. plantarum* E51 or LGG in MRS broth and declined thereafter (Fig. 1). The maximum growth of *C. difficile* was slightly decreased by co-incubation with E51, compared to that of co-culture with LGG (Fig. 1).

### *L. plantarum* E51 attenuated *C. difficile*-induced damage to Caco-2 monolayer integrity

**Fig. 2:**
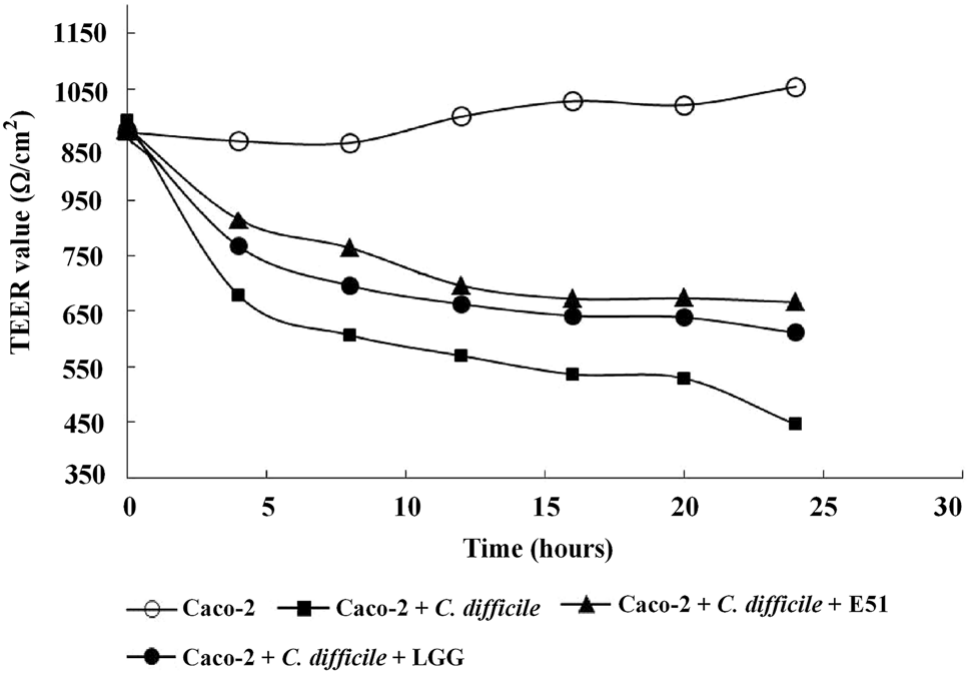
Effects of *L. plantarum* E51 and LGG on *C. difficile*-damaged transepithelial electrical resistance of Caco-2 cell monolayer. Caco-2 monolayers were incubated with or without *C. difficile* in the presence or absence of *L. plantarum* E51 or LGG for 24 h. Only one measurement was made at each time point

TEER values of Caco-2 cells remained relatively steady over the 24 h incubation (Fig. 2). The integrity of the Caco-2 monolayer was disrupted in the presence of *C. difficile*, resulting in a gradual decrease in TEER. Both *L. plantarum* E51 and LGG attenuated the *C. difficile*-induced decrease in TEER, suggesting protective effects on Caco-2 cell membrane tight junctions or the inhibition of toxin production by *C. difficile*. This protective effect was greater for *L. plantarum* E51 than LGG (Fig. 2).

### *L. plantarum* E51 reversed *C. difficile*-evoked changes in IL-8 secretion and claudin-1 protein expression

**Fig. 3:**
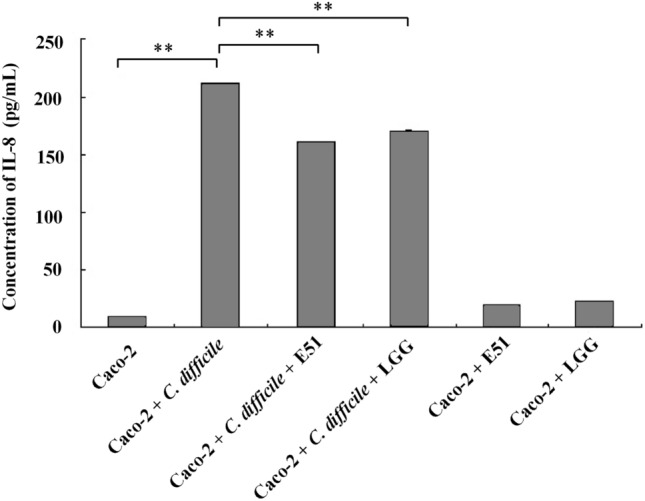
Effects of *L. plantarum* E51 and LGG on *C. difficile-*induced IL-8 production by Caco-2 cells. Caco-2 monolayers were treated with or without *C. difficile* in the presence or absence of *L. plantarum* E51 or LGG for 15 h. Data are presented as the mean ± SD of triplicate samples. **p < 0.01

**Fig. 4:**
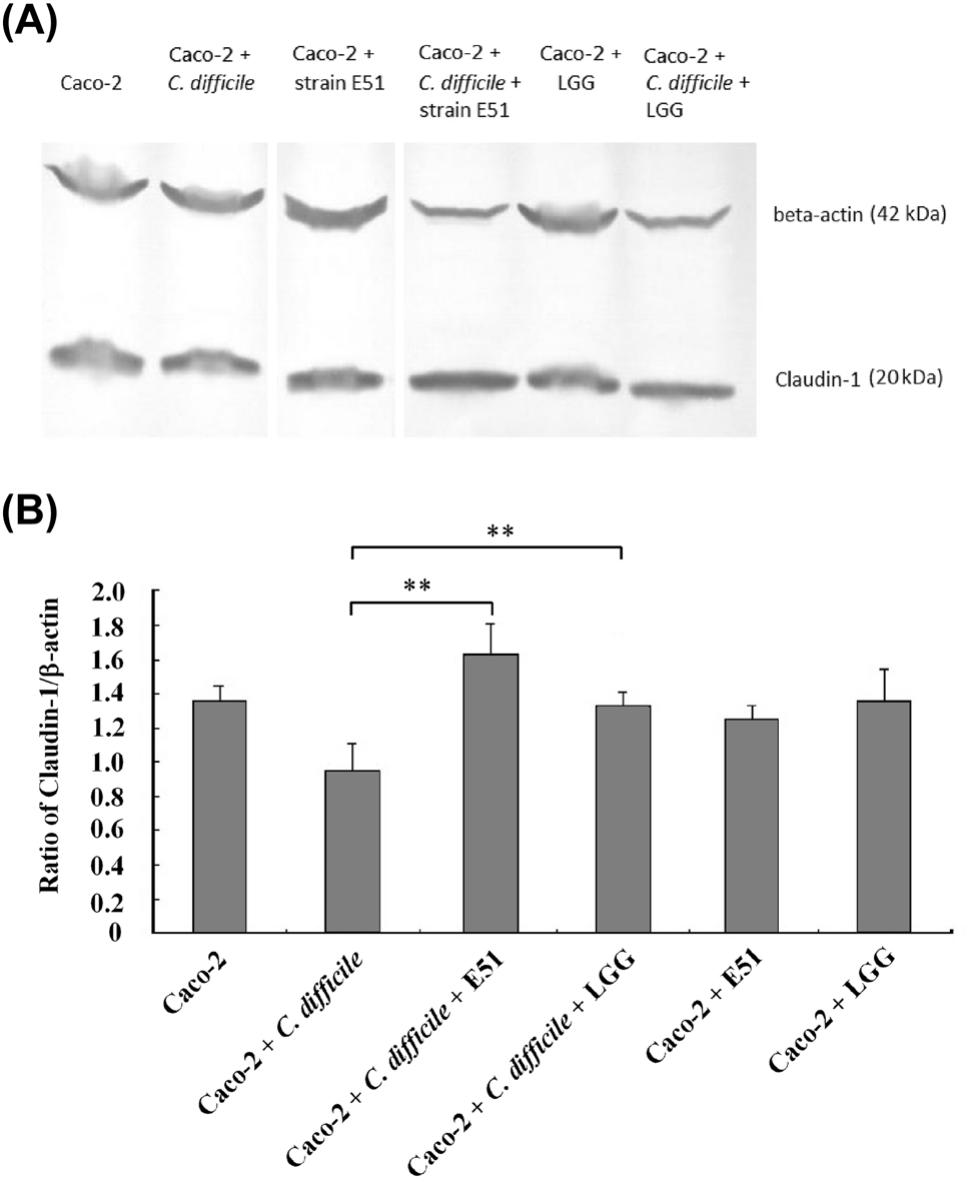
Effects of *L. plantarum* E51 and LGG on *C. difficile-*mediated inhibition of claudin-1 protein expression in Caco-2 cells. Caco-2 monolayers were treated with or without *C. difficile* in the presence or absence of *L. plantarum* E51 or LGG for 15 h. **A** The representative Western blot images. **B** Histogram of western blot results. Data are presented as the mean ± SD of triplicate samples. **p < 0.01

*Clostridioides difficile* significantly increased IL-8 secretion by Caco-2 cells (*p* < 0.01). In the presence of *L. plantarum* E51 or LGG, this upregulative effect of *C. difficile* on IL-8 secretion was significantly suppressed (both *p* < 0.01) (Fig. 3). On the other hand, *C. difficile* inhibited the protein expression of claudin-1 in Caco-2 cells. Both *L. plantarum* E51 and LGG reversed the inhibitory effect of *C. difficile* on claudin-1 expression, and significantly upregulated claudin-1 protein expression (both *p* < 0.01) (Fig. 4).

### *L. plantarum* E51 inhibited *C. difficile* adhesion to Caco-2 cells

**Fig. 5:**
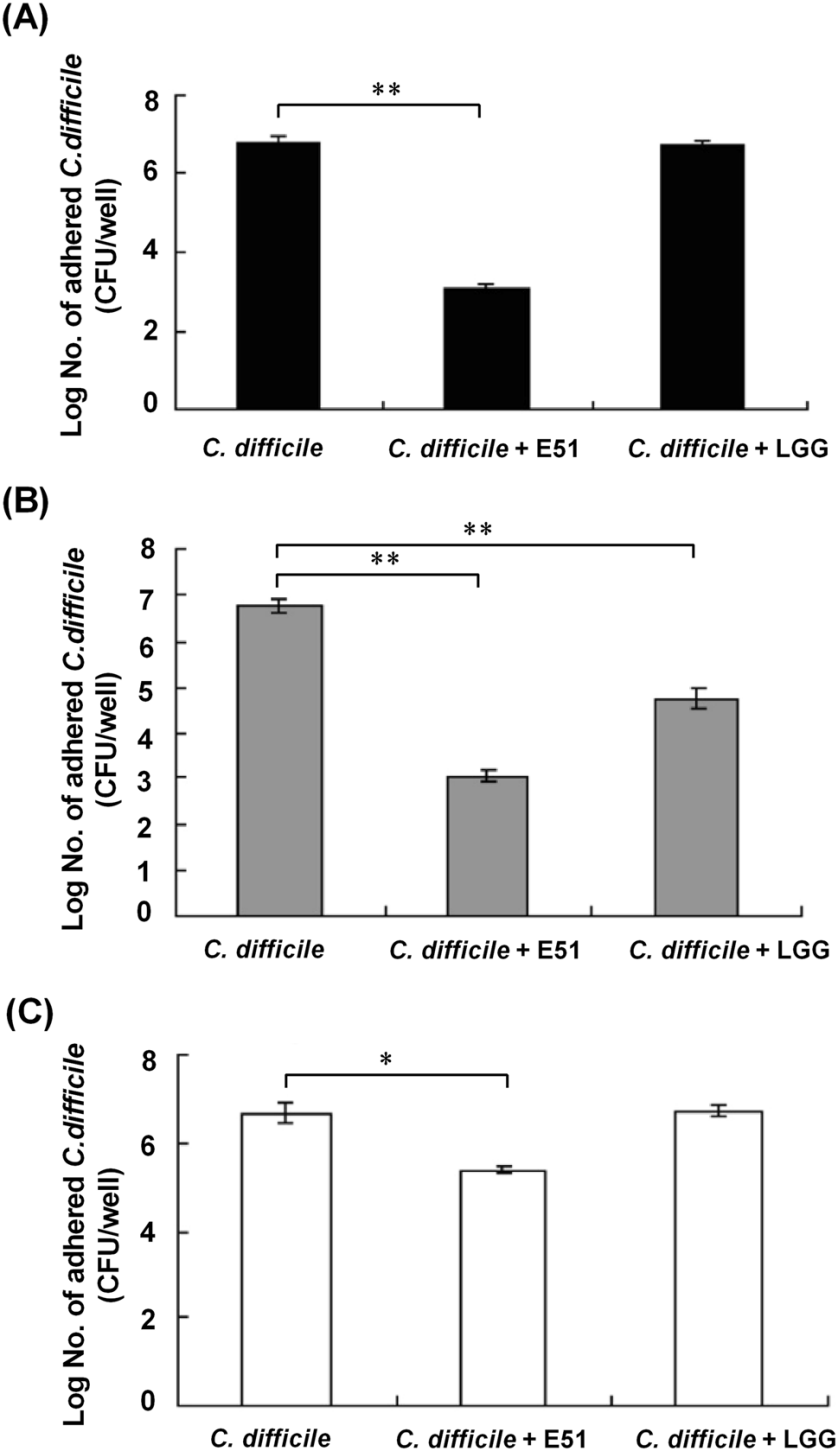
Effects of *L. plantarum* E51 and LGG on the capacity of *C. difficile* to adhere to Caco-2 cells. **A** Competition assay. **B** Exclusion assay. **C** Displacement assay. Data are presented as the mean ± SD of triplicate samples. **p < 0.01; *p < 0.05

The effect of *L. plantarum* E51 on the capacity of *C. difficile* to adhere to Caco-2 cells was evaluated using competition, exclusion, and displacement assays. In the competition assay, *L. plantarum* E51, but not LGG, significantly reduced the number of *C. difficile* adhered to Caco-2 cells (*p* < 0.01) (Fig. 5A). In the exclusion assay, both *L. plantarum* E51 and LGG significantly decreased the number of *C. difficile* adhered to Caco-2 cells (both *p* < 0.01) (Fig. 5B). The displacement assay showed that *L. plantarum* E51 significantly decreased the number of *C. difficile* adhered to Caco-2 cells (*p* < 0.01) (Fig. 5C).

## Discussion

In this preliminary in vitro study, we observed that *L. plantarum* E51 exerted a variety of protective effects against *C. difficile* in Caco-2 cells**.** *L. plantarum* E51 tolerated low pH and high-concentration bile salts. Compared to LGG, *L. plantarum* E51 exhibited better inhibition of *C. difficile* growth. In addition, *L. plantarum* E51 attenuated *C. difficile*-induced damage to Caco-2 monolayers, enhanced claudin-1 protein expression, suppressed *C. difficile-*induced IL-8 secretion, and decreased the adhesion of *C. difficile* to Caco-2 cells. Together, *L. plantarum* E51 substantially protected against *C. difficile*-induced damages on Caco-2 intestinal barrier functions.

*C. difficile* Toxins A and B disrupt the actin cytoskeleton and tight junctions of intestinal epithelial cells (Czepiel et al. 2019), allowing the toxins to enter the laminar propria and submucosa, where they induce the production of proinflammatory and cytotoxic molecules, such as IL-8 (Boonma et al. 2014). Increased IL-8 secretion by intestinal epithelial cells causes a massive influx of neutrophils into the colonic mucosa, resulting in epithelial damage due to inflammatory edema (Rupnik et al. 2009). We observed multiple protective effects of *L. plantarum* E51 on *C. difficile* infection, suggesting that the mechanisms underlying the protective effects may involve multiple steps in *C. difficile* pathogenesis. Our findings suggest that *L. plantarum* E51 may inhibit *C. difficile* infection in at least three ways: (i) suppressing *C. difficile* growth, (ii) preserving Caco-2 monolayer integrity via upregulating tight junction protein, and (iii) inhibiting *C. difficile* adhesion to Caco-2 cells.

The distribution of mechanisms underlying probiotic effects shows that many are widespread among species, while others are species specific (Hill et al. 2014). Our proposed mechanism for the effects of *L. plantarum* E51 on *C. difficile* is supported by observations in other bacterial species. Yu et al. reported that *Lactobacillus delbrueckii* ssp. *delbrueckii* D11 and *Levilactobacillus brevis* inhibit the adhesion of pathogenic bacteria to Caco-2 cells and concluded that these strains may be useful for protecting against pathogenic infection (Yu et al. 2011). Similarly, *Lactobacillus delbrueckii* ssp. *bulgaricus* B-30892 was reported to inhibit the adhesion of *C. difficile* to Caco-2 cells (Banerjee et al. 2009). Upregulation of claudin-1 was observed to reverse the effect of aspirin-induced epithelial barrier disruption to improve the epithelial barrier function in vitro (Nishii et al. 2020). Another potential mechanism that was not investigated in this study was observed in several strains of *Bifidobacterium spp.* and *Lactiplantibacillus spp*., which exerted antagonistic effects on the production of *C. difficile* toxins A and B (Trejo et al. 2010).

Our observation that *L. plantarum* E51 decreased *C. difficile*-induced secretion of IL-8 is consistent with the finding that *L. rhamnosus* L34 and *L. casei* L39 suppressed *C. difficile-*induced IL-8 production and inflammation (Boonma et al. 2014). Another *L. planatarum* species*,* Inducia (DSM 21379), was shown to decrease hamster gut colonization by *C. difficile *in vivo (Rätsep et al. 2017), and a combination of *Lactiplantibacillus and Bifidobacterium* strains conferred protection against *C. difficile* infection in antibiotic-treated mice (Kondepudi et al. 2014).

Because numerous mechanisms underlie the probiotic effects of studied species, differences in the type and extent of effects exerted by LGG and *L. plantarum* E51 on *C. difficile* are expected. LGG, a confirmed probiotic species, was widely used as a control strain to gauge the effects of *L. plantarum* E51 in this study*.* While LGG and *L. plantarum* E51 were similar overall in the types of effects exerted, the extent of these effects differed in some respects. Significant differences between LGG and *L. plantarum E51* were observed in competition and displacement assays for inhibition of *C. difficile* adhesion to Caco-2 cells, with *L. plantarum* E51 demonstrating relatively strong inhibition.

Furthermore, an inhibitory effect of lactic acid produced by *L. plantarum* strain Inducia (DSM 21379) on the growth of *C. difficile* has been previously reported (Rätsep et al. 2017); however, the authors also suggest that this factor may not be the only mechanism underlying the observed inhibitory effect. In the present study, the pH values of the culture medium were not substantially decreased during the growth of LGG or *L. plantarum* E 51. Hence, the effect of lactic acid production by LGG and *L. plantarum* E51 in the inhibition of *C. difficile* growth appears to be minor in the present study.

This study has several limitations. First of all, since *C. difficile* Toxins A and B were not quantified in this study, the possibility that cellular damage by *C. difficile* observed may be at least in part caused by its produced toxins cannot be ruled out. In addition, because of aerobic respiration, Caco-2 cells constantly require oxygen. Hence, the experiments involving Caco-2 cells had to be performed in aerobic condition in the present study; however, the extent to which such aerobic condition affects the vitality and virulence of *C. difficile* is unclear*.* Therefore, although the Caco-2 monolayer model has been extensively used in the investigation *C. difficile* infection, in vivo studies are still warranted to assess the effectiveness of candidate probiotic agents against *C. difficile* infection.

In conclusion, the findings of this in vitro study demonstrated that *L. plantarum* E51 effectively reduced the harmful effects of *C. difficile*, thereby preserving Caco-2 intestinal barrier functions. Although *L. plantarum* E51 exhibited several necessary attributes of probiotic agents, further in vivo studies are warranted to investigate the probiotic potential of *L. plantarum* E51 against *C. difficile* infection.
